# Prognostic role of statins in colorectal cancer: a systematic review and meta-analysis

**DOI:** 10.3389/fonc.2026.1763323

**Published:** 2026-03-18

**Authors:** Gang Li, Wenzhong Zhang, Jie Wang, Baiying Xu, Yongbing Wang

**Affiliations:** Department of General Surgery, Shanghai Pudong New Area People’s Hospital, Shanghai, China

**Keywords:** colorectal cancer, meta-analysis, prognosis, statins, systematic review

## Abstract

**Background:**

Colorectal cancer (CRC) is a leading cause of global cancer incidence and mortality. While the anti-tumor potential of statins has gained increasing attention, their exact impact on patient prognosis remains controversial. This systematic review and meta-analysis aims to comprehensively assess the association between statin use and survival outcomes in patients with CRC.

**Methods:**

We systematically searched the PubMed, Embase, Cochrane Library, and Web of Science databases for studies published from inception until October 31, 2025, that compared the impact of statin use versus non-use on the prognosis of patients with CRC. The quality of the included studies was assessed using the Newcastle-Ottawa Scale. The effect of statins was measured using hazard ratios (HRs) with 95% confidence intervals (CIs), and a random-effects model was employed for all pooled analyses.

**Results:**

A total of 25 observational studies involving 179, 979 CRC patients were included. Statin use was significantly associated with reduced ACM (HR: 0.80; 95%CI: 0.74-0.86; P < 0.001) and CSM (HR: 0.77; 95%CI: 0.73-0.81; P < 0.001) in CRC patients. These benefits were consistently observed in both pre-diagnosis (ACM: HR = 0.78; CSM: HR = 0.78) and post-diagnosis statin use (ACM: HR = 0.83; CSM: HR = 0.75). However, no significant association was found between statin use and DFM (HR: 0.88; 95%CI: 0.60-1.29; P = 0.513) or RFM (HR: 1.01; 95%CI: 0.94-1.09; P = 0.831).

**Conclusion:**

Statin use is associated with a significant reduction in ACM and CSM among CRC patients. This benefit is consistently observed with both pre-diagnosis and post-diagnosis use, suggesting statins may serve as a potential intervention to improve prognosis in CRC patients.t

**Systematic Review Registration:**

https://inplasy.com/, identifier INPLASY2025110064.

## Introduction

Colorectal cancer (CRC) is a major disease that poses a serious threat to human health worldwide, with its incidence and mortality rates ranking among the highest of all malignant tumors. According to the GLOBOCAN 2020 data, there were 1.93 million new cases of CRC globally and approximately 935, 000 deaths, accounting for 10.0% of all new cancer cases and 9.4% of all cancer-related deaths, respectively (1). The high incidence and mortality rates of CRC impose a heavy burden on patients’ families and society as a whole (2). Despite significant advances in comprehensive treatment strategies such as surgery, chemotherapy, radiotherapy, and targeted therapy, the prognosis of CRC patients still varies widely (3–6). Identifying factors that can improve prognosis remains an important direction in clinical practice.

Recently, drug repurposing—the application of existing drugs to treat new diseases—has demonstrated significant potential in the field of oncology. Among such drugs, statins, widely used as first-line agents for both primary and secondary lipid-lowering therapy in cardiovascular diseases (7), have attracted considerable attention due to their potential antitumor effects (8). Substantial evidences indicates that statins may influence tumor biology through multiple mechanisms, including inhibiting cell proliferation, inducing apoptosis, impeding tumor invasion and metastasis, and enhancing chemosensitivity by modulating the tumor microenvironment (9, 10). These effects are primarily attributed to their inhibition of the mevalonate pathway, which subsequently disrupts the prenylation of small GTPases such as Ras and Rho (11).

Studies have explored the association between statin use before and after diagnosis and the prognosis of CRC patients. However, the conclusions drawn from these studies are inconsistent and even contradictory. Although meta-analyses have previously examined the impact of statin use on the prognosis of CRC patients (12–14), a large number of relevant studies have been published in recent years, necessitating an update of the findings. Therefore, this study aims to conduct a comprehensive systematic review and meta-analysis of the existing evidence to provide a clear evaluation of the effect of statin use on the prognosis of CRC patients.

## Methods

### Data sources, search strategy, and selection criteria

This study was conducted in accordance with the Preferred Reporting Items for Systematic Reviews and Meta-Analyses (PRISMA) statement (15). Using systematic review and meta-analysis methods, it comprehensively integrates and evaluates relevant research on the association between statin use and the prognosis of CRC patients. The study protocol has been registered on the INPLASY platform under registration number INPLASY2025110064.

We systematically searched the PubMed, Embase, Cochrane Library, and Web of Science databases up to October 31, 2025, using a combination of subject headings and free-text terms. The core search terms included: “statin” OR “statins” AND (“colorectal cancer” OR “rectal cancer” OR “colon cancer”) AND “prognosis”. Detailed search strategies for each database are provided in **Supplementary File 1**. Additionally, we manually screened the reference lists of included studies to avoid missing relevant literature.

Two authors independently performed the literature screening, with any discrepancies resolved through discussion until a consensus was reached. Studies were included according to the following criteria: (1) Study population: patients with pathologically confirmed CRC; (2) Exposure: use of statins (either before or after CRC diagnosis); (3) Control: no use of statins; (4) Outcome: reporting of at least one of the following prognostic indicators—all-cause mortality (ACM), cancer-specific mortality (CSM), disease-free mortality (DFM), or recurrence-free mortality (RFM); (5) Study type: observational studies such as cohort or case-control studies, or randomized controlled trials (RCTs). Exclusion criteria were as follows: (1) basic research, reviews, case reports, conference abstracts, or commentaries; (2) study populations with colorectal precancerous lesions (e.g., adenomas) or other concurrent malignancies; (3) studies that did not specify statin use status or lacked available prognostic data; and (4) duplicate publications or studies with overlapping data.

### Data collection and quality assessment

Two investigators independently extracted data using a standardized form, which included the first author, year of publication, country where the study was conducted, sample size, age, sex, tumor location, tumor stage, timing of statin use, information on statin type, dose, or duration if available, treatment regimen, reported outcomes, and adjusted confounding variables. Any discrepancies were resolved through discussion or, when necessary, by a third investigator. In addition, the included studies were assessed using the Newcastle–Ottawa Scale (NOS), which evaluates three domains: selection of study populations, comparability of groups, and outcome assessment. Studies with scores ≥7 were considered high quality, those scoring 4–6 were deemed moderate quality, and studies with scores ≤3 were classified as low quality (16). The quality assessment was performed independently by two investigators, with disagreements resolved by a third investigator through referral to the original publication.

### Statistical analysis

Hazard ratios (HRs) with 95% confidence intervals (CIs) were used as effect measures to analyze the associations between statin use and ACM, CSM, DFM, and RFM in CRC patients. All analyses were performed using random-effects models, as this approach accounts for potential variability between studies (17, 18). Heterogeneity among studies was assessed using the I² statistic and the Q-test p-value. Significant heterogeneity was considered present when I² ≥ 50% or p < 0.10 (19, 20). To evaluate the stability of the pooled results, we conducted a leave-one-out sensitivity analysis by sequentially excluding individual studies and recalculating the pooled effect size to observe whether the results changed substantially (21). Subgroup analyses were performed for ACM and CSM based on study design, country, mean patient age, tumor site, extent of covariate adjustment, and study quality. Differences between subgroups were compared using interaction tests (22). Although our inclusion criteria allowed for RCTs, all eligible studies identified were observational (cohort or case-control). To assess the potential impact of study design on our findings, we conducted subgroup analyses for ACM and CSM by categorizing studies as prospective or retrospective. Publication bias was initially assessed visually using funnel plots and further evaluated statistically with Egger’s and Begg’s tests (23, 24). If publication bias was detected, the trim-and-fill method was applied to adjust for its potential impact (25). All tests were two-sided, with a significance level set at 0.05. Statistical analyses were conducted using STATA 15.0 (StataCorp, College Station, TX, USA).

## Results

### Literature search

**Figure 1:**
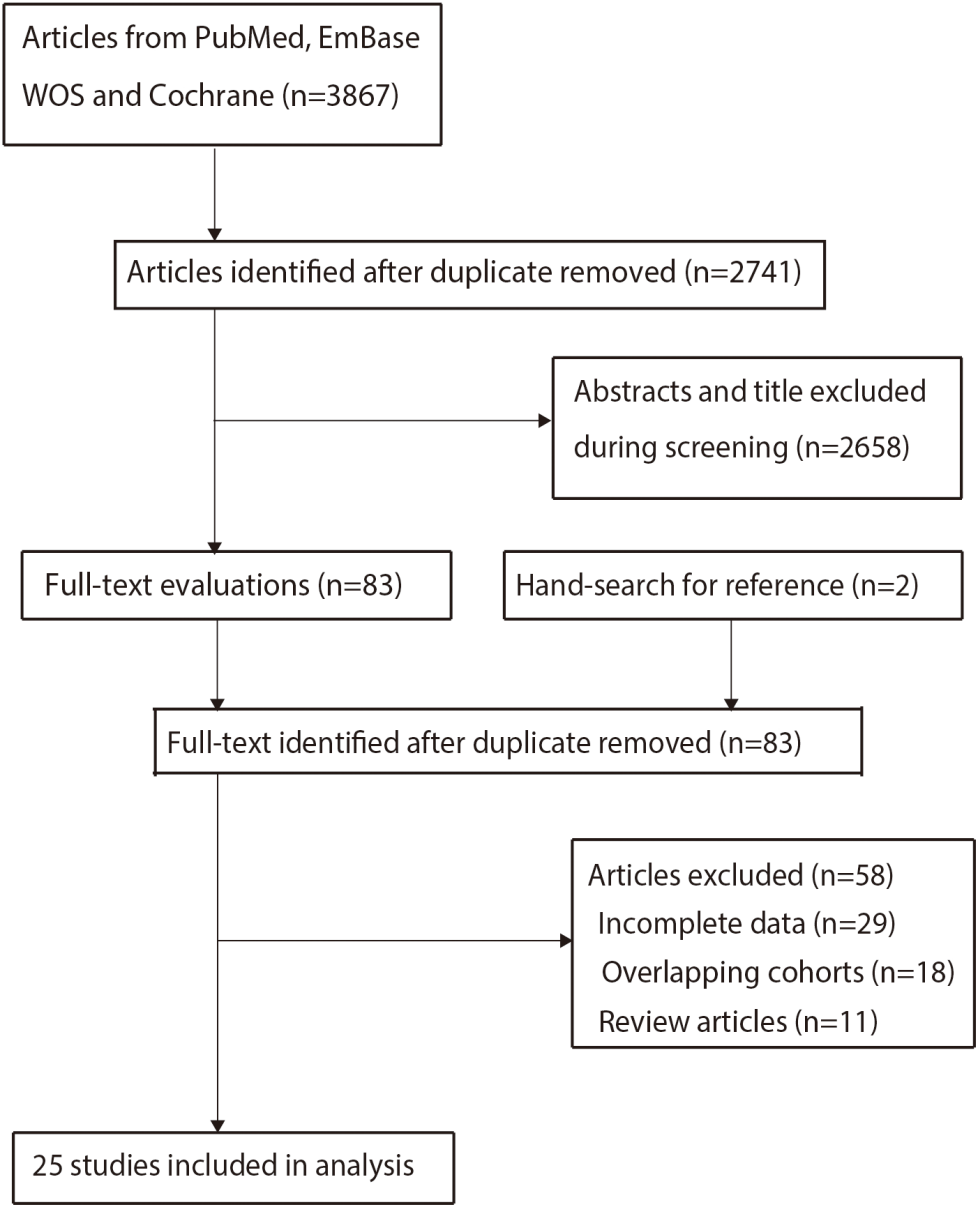
PRISMA flow diagram of the literature selection process. This flow diagram follows the Preferred Reporting Items for Systematic Reviews and Meta-Analyses (PRISMA) statement guidelines, illustrating the literature selection process of this study.

Our initial search identified 3, 867 potentially relevant records. After removing duplicates, 2, 741 records remained. Through title and abstract screening, we excluded 2, 658 records, leaving 83 articles for full-text review. Of these, 58 studies were excluded for the following reasons: lack of prognostic outcomes (n=29), reporting on overlapping cohorts (n=18), or being review articles (n=11). Ultimately, 25 studies met the inclusion criteria and were included in the analysis (26–50). The detailed literature selection process is illustrated in **Figure 1**.

### Study characteristics

The included studies were published between 2009 and 2024, comprising 9 prospective and 16 retrospective studies. Geographically, the studies covered Asia (5 studies), Europe (17 studies), and North America (3 studies). The total sample size was 179, 979 CRC patients, with individual study sample sizes ranging from 105 to 43, 487 patients. Detailed characteristics of the included studies are presented in **Table 1**. All studies clearly documented statin use. Specifically, 16 studies reported statin use before CRC diagnosis, while 14 studies reported statin use after diagnosis. Details regarding specific statin types, doses, or durations of use, were not reported in the original studies. Studies defined exposure broadly as ‘statin use’ without granular pharmacological details. Quality assessment using the NOS showed that 5 studies scored 8 points, 11 studies scored 7 points, and the remaining 9 studies scored 6 points.

**Table 1:** Baseline characteristics of included studies and involved patients.

Study	Study design	Country	Sample size	Age (years)	Male (%)	Cancer site	Cancer stage	Initiation of statin use	Treatments	Reported outcomes	Adjusted factors	NOS
Siddiqui 2009 (26)	Retrospective	USA	1, 309	67.2	100.0	CRC	I-IV	Pre	S+C/R	CSM	BMI and NSAIDs	6
Ng 2011 (27)	Prospective	USA	842	59.8	56.3	Colon	III	Post	S+C	ACM, DFM, RFM	Age, sex, family history of CRC, baseline performance status, depth of invasion through bowel wall, number of positive lymph nodes, perineural invasion, Extravascular invasion, Postoperative CEA, treatment arm, BMI, PA, Western pattern diet, and consistent aspirin use	8
Lakha 2012 (28)	Prospective	UK	603	60.7	53.2	CRC	I-IV	Pre, Post	S+C/R	ACM, CSM	Age, sex and AJCC stage	7
Nielsen 2012 (29)	Prospective	Denmark	43, 487	69.0	NA	Colon	I-IV	Pre	S+C/R	CSM	Age, stage, chemotherapy, radiotherapy, CVD before cancer, DM before cancer, birth year, sex, descent, education level, and residential area	8
Mace 2013 (30)	Retrospective	USA	407	59.4	71.7	Rectal	I-IV	Post	S+C/R	ACM, CSM, DFM, RFM	Age, BMI, ASA class III/IV, and pathological stage	6
Zanders 2013 (31)	Prospective	Netherlands	287	NA	NA	CRC	I-IV	Pre	S+C/R	ACM	NA	7
Ma 2013 (32)	Retrospective	China	9, 950	NA	NA	CRC	I-II	Pre	S+C	ACM, CSM	NA	7
Ishikawa 2014 (33)	Retrospective	Japan	742	68.6	54.7	CRC	I-IV	Pre	S+C/R	ACM, DFM	Crude	6
Cardwell 2014 (34)	Prospective	UK	7, 657	≥ 70.0	55.3	CRC	I-III	Pre, Post	S+C/R	ACM, CSM	Year of diagnosis, age, sex, stage, surgery/radiotherapy/chemotherapy within 6 months, site, comorbidities, and other medication use after diagnosis as timevarying covariates, grade, deprivation, and smoking before diagnosis	8
Krens 2014 (35)	Retrospective	Netherlands	529	62.7	58.4	CRC	IV	Post	C	ACM, RFM	Age, prior adjuvant therapy, aspirin use, >1 organ affected by metastatic spread, treatment arm, KRAS mutation status, and a KRAS*statin	7
Hoffmeister 2015 (36)	Prospective	Germany	2, 697	68.0	59.8	CRC	I-IV	Post	S+C/R	ACM, CSM, RFM	Age at diagnosis, sex, Cancer stage, location of CRC, surgery, neoadjuvant treatment, chemotherapy, radiotherapy, BMI, smoking, average lifetime PA, use of NSAIDs, ever use of HRT(women), previous large bowel endoscopy, DM, hyperlipidemia, MI, stroke, heart failure, participation in general health check-ups, and for a time-dependent effect of chemotherapy	8
Kim 2015 (37)	Retrospective	Korea	686	59.1	40.8	CRC	III	Post	S+C/R	CSM, DFM	Age, sex, comorbidity, pre-diagnosis aspirin use, medication, cancer site, initial stage, pathological differentiation	6
Shao 2015 (38)	Prospective	China	17, 115	66.6	57.0	CRC	I-III	Pre	S+C/R	ACM, CSM	Age, sex, diagnosis year, physician visits and hospitalization 1 y before diagnosis, exposure to aspirin, other NSAIDs, insulin, oral antidiabetic drugs, angiotensin-converting enzyme inhibitors, and angiotensin II receptor blockers, and the aforementioned comorbidities	7
Gray 2016 (39)	Prospective	UK	8, 391	≥ 70.0	55.7	CRC	I-III	Pre, Post	S+C/R	ACM, CSM	Age, sex, year of diagnosis, deprivation, site, comorbidities and aspirin use, stage, grade, and cancer treatment within 6 months	8
Gray 2017 (40)	Retrospective	UK	680	≥ 70.0	54.4	Colon	II-III	Post	S+C	ACM, CSM	Age, gender, year of diagnosis, grade, MSI status, ECOG performance status, family history of CRC, adjuvant chemotherapy use, stage, and aspirin use, Charlson Comorbidity Index score	7
Lash 2017 (41)	Prospective	Denmark	21, 152	≥ 70.0	52.9	CRC	I-III	Post	S+C/R	ACM, CSM, RFM	Age at colorectal cancer diagnosis, sex, calendar period of diagnosis, AJCC stage at diagnosis, surgical urgency, receipt of neoadjuvant or adjuvant chemotherapy, receipt of radiation therapy, Charlson comorbidity score at diagnosis, and history of inflammatory bowel disease at diagnosis	7
Voorneveld 2017 (42)	Retrospective	Netherlands	999	≥ 70.0	50.6	Colon	I-IV	Post	S+C	ACM, CSM	Sex, age, comorbidity, year of incidence, histological grade, stage, microsatellite status, chemotherapy, and aspirin use	7
Kotti 2019 (43)	Retrospective	Sweden	465	68.9	57.2	Rectal	I-IV	Pre	S+C/R	ACM, CSM, DFM	Sex, stage, neoadjuvant/adjuvant chemotherapy, history of coronary artery disease, history of DM, history of stroke	6
Pourlotfi 2020 (44)	Retrospective	Sweden	11, 966	68.0	60.4	Rectal	I-IV	Pre	S+C	ACM, CSM	Age, sex, ASA classification, CCI, cancer stage, surgical procedure, surgical technique, neoadjuvant therapy, adjuvant therapy	7
Han 2021 (45)	Retrospective	Korea	341	NA	NA	CRC	I-IV	Post	S+C/R	ACM	CCI, sex, age, BMI, residence area, income, year of diagnosis	6
Pourlotfi 2022 (46)	Retrospective	Sweden	19, 118	72.3	48.0	Colon	I-III	Pre	S+C	ACM, CSM	Age, sex, ASA classification, CCI, cancer stage, received adjuvant therapy, surgical technique, type of surgical resection, and year of surgery	7
Erkinantti 2022 (47)	Retrospective	Finland	1, 271	74.0	60.0	Rectal	I-IV	Pre, Post	S+C/R	CSM	Age, year, duration of DM, and cancer stage	6
Erkinantti 2022 (48)	Retrospective	Finland	2, 252	75.0	49.0	Colon	I-IV	Pre, Post	S+C/R	CSM	Age, year, duration of DM, and cancer stage	6
Loffler 2024 (49)	Retrospective	Denmark	26, 928	NA	55.4	CRC	I-III	Pre	S+C/R	ACM, DFM	Age, sex, BMI, CCI, ECOG, ASA, UICC stage, tumor localization	7
Erdat 2024 (50)	Retrospective	Turkey	105	66.0	57.1	CRC	II-IV	Pre	C	ACM, DFM	Age, sex, primary location, cancer stage, relapsed, metastatic sites, RAS mutant, RAF mutant, comorbid diseases, previous treatment lines	6

### All-cause mortality

**Figure 2:**
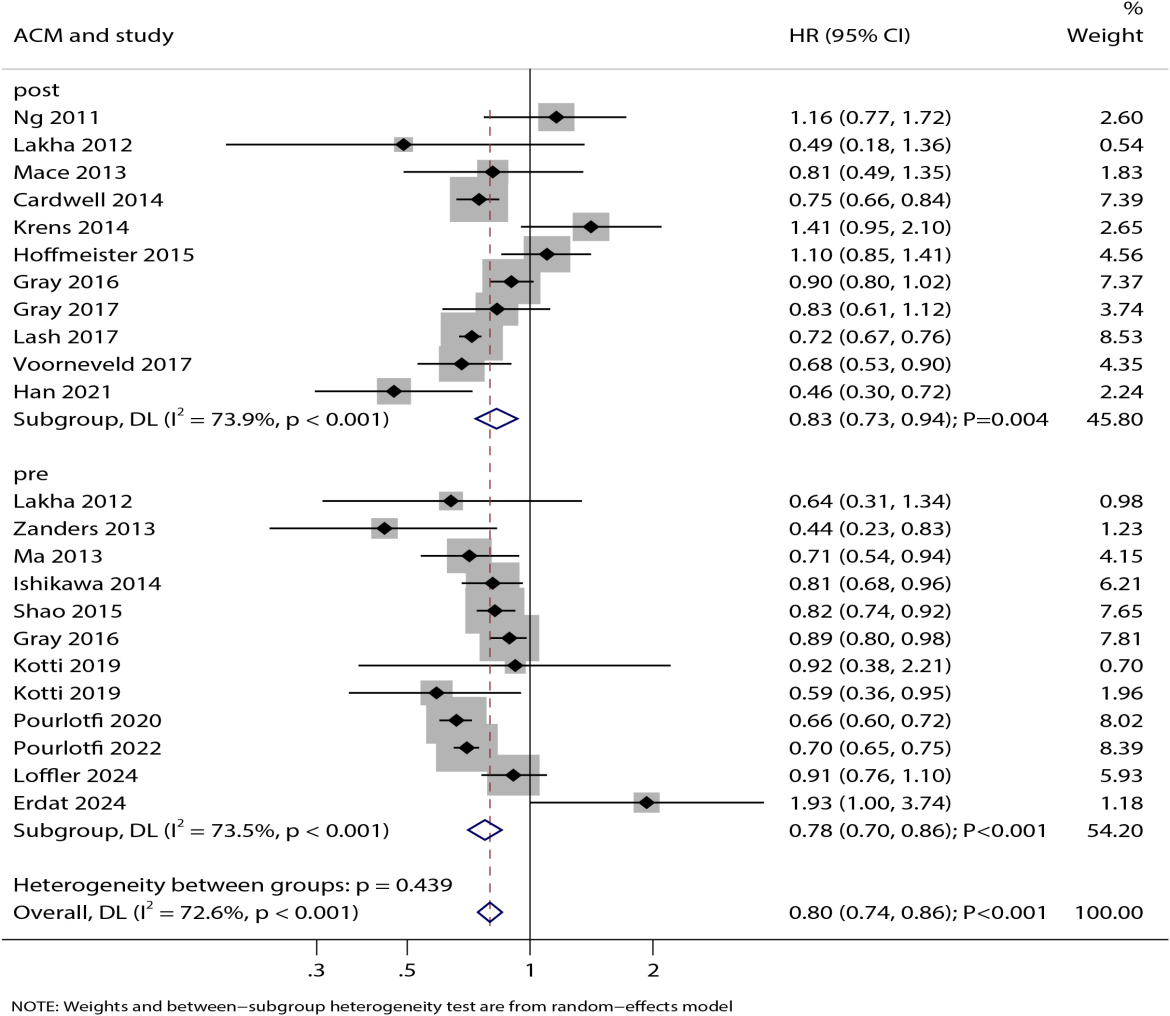
Forest plot of the association between statin use and all-cause mortality (ACM) in patients with colorectal cancer. The size of each square represents the study weight, the horizontal line indicates the 95%CI, the vertical line is the null effect line (HR = 1), and the diamond represents the pooled effect size with 95%CI.

The association between statin use and ACM was reported in 11 studies for both pre-diagnosis and post-diagnosis use (**Figure 2**). Our analysis showed that statin use was significantly associated with improved ACM (HR: 0.80; 95% CI: 0.74–0.86; P < 0.001). This benefit was observed both for pre-diagnosis statin use (HR: 0.78; 95% CI: 0.70–0.86; P < 0.001) and post-diagnosis statin use (HR: 0.83; 95% CI: 0.73–0.94; P = 0.004). Significant heterogeneity was detected for both pre-diagnosis (I² = 73.5%; P < 0.001) and post-diagnosis statin use (I² = 73.9%; P < 0.001). Sensitivity analysis indicated that the pooled estimates for the effect of statin use on ACM—both before and after diagnosis—were not driven by any single study (**Supplementary File 2**).

Subgroup analysis revealed that pre-diagnosis statin use was significantly associated with improved ACM in all subgroups except those with moderate-quality studies. In contrast, post-diagnosis statin use showed no significant effect on ACM in retrospective cohorts, studies with mean age <70 years, studies including specific rectal or colon cancer populations, and low-quality study cohorts (**Table 2**). The association between pre-diagnosis statin use and ACM was modified by cancer site (P = 0.003), while the association for post-diagnosis use was influenced by geographic region (P = 0.010) and mean patient age (P = 0.001). No significant publication bias was detected for the association of statin use with ACM, either for pre-diagnosis (Egger’s test P = 0.574; Begg’s test P = 0.945) or post-diagnosis use (Egger’s test P = 0.322; Begg’s test P = 1.000) (**Supplementary File 3**).

**Table 2:** Subgroup analyses for ACM and CSM.

Outcomes	Factors	Subgroups	HR and 95%CI	*P value*	*I^2^* (%)	Q statistic	Interaction test
Prediagnosis statin use and ACM	Study design	Prospective	0.82 (0.72-0.94)	0.005	48.5	0.121	0.381
		Retrospective	0.76 (0.67-0.86)	< 0.001	67.4	0.003	
	Country	Western	0.77 (0.66-0.89)	< 0.001	78.8	< 0.001	0.594
		Eastern	0.81 (0.74-0.88)	< 0.001	0.0	0.636	
	Mean age (years)	≥ 70.0	0.79 (0.62-1.00)	0.046	93.0	< 0.001	0.939
		< 70.0	0.78 (0.66-0.92)	0.003	69.8	0.003	
	Cancer sites	CRC	0.84 (0.75-0.93)	0.001	50.7	0.048	0.003
		Rectal	0.66 (0.60-0.72)	< 0.001	0.0	0.687	
		Colon	0.70 (0.65-0.75)	< 0.001	–	–	
	Adjusted level	High	0.80 (0.70-0.90)	< 0.001	81.5	< 0.001	0.508
		Low-moderate	0.80 (0.68-0.95)	0.009	0.0	0.539	
	Study quality	High	0.76 (0.68-0.85)	< 0.001	78.0	< 0.001	0.422
		Moderate	0.90 (0.60-1.36)	0.622	63.9	0.040	
Postdiagnosis statin use and ACM	Study design	Prospective	0.85 (0.73-0.98)	0.028	78.5	< 0.001	0.682
		Retrospective	0.79 (0.57-1.08)	0.141	73.4	0.005	
	Country	Western	0.85 (0.75-0.97)	0.016	72.9	< 0.001	0.010
		Eastern	0.46 (0.30-0.72)	0.001	–	–	
	Mean age (years)	≥ 70.0	0.77 (0.70-0.86)	< 0.001	64.3	0.025	0.001
		< 70.0	1.08 (0.87-1.35)	0.478	26.3	0.246	
	Cancer sites	CRC	0.83 (0.70-0.97)	0.022	81.9	< 0.001	0.991
		Rectal	0.81 (0.49-1.34)	0.415	–	–	
		Colon	0.84 (0.63-1.12)	0.232	58.1	0.092	
	Adjusted level	High	0.87 (0.76-0.99)	0.037	78.4	< 0.001	0.061
		Low-moderate	0.58 (0.39-0.86)	0.007	27.1	0.254	
	Study quality	High	0.86 (0.75-0.98)	0.024	75.9	< 0.001	0.222
		Moderate	0.60 (0.35-1.04)	0.071	61.9	0.105	
Prediagnosis statin use and CSM	Study design	Prospective	0.82 (0.78-0.85)	< 0.001	0.0	0.436	0.031
		Retrospective	0.73 (0.67-0.80)	< 0.001	14.1	0.320	
	Country	Western	0.78 (0.74-0.83)	< 0.001	38.1	0.095	0.651
		Eastern	0.76 (0.68-0.85)	< 0.001	0.0	0.658	
	Mean age (years)	≥ 70.0	0.83 (0.78-0.87)	< 0.001	0.0	0.480	0.071
		< 70.0	0.73 (0.67-0.81)	< 0.001	31.6	0.187	
	Cancer sites	CRC	0.81 (0.76-0.86)	< 0.001	16.3	0.309	0.205
		Rectal	0.66 (0.53-0.82)	< 0.001	34.0	0.208	
		Colon	0.79 (0.74-0.83)	< 0.001	0.0	0.630	
	Adjusted level	High	0.78 (0.73-0.84)	< 0.001	50.8	0.047	0.829
		Low-moderate	0.77 (0.70-0.86)	< 0.001	0.0	0.601	
	Study quality	High	0.79 (0.74-0.84)	< 0.001	40.5	0.108	0.595
		Moderate	0.76 (0.67-0.86)	< 0.001	13.2	0.330	
Postdiagnosis statin use and CSM	Study design	Prospective	0.81 (0.68-0.95)	0.010	70.8	0.008	0.140
		Retrospective	0.68 (0.59-0.79)	< 0.001	1.5	0.406	
	Country	Western	0.76 (0.68-0.85)	< 0.001	53.3	0.023	0.202
		Eastern	0.48 (0.24-0.96)	0.039	–	–	
	Mean age (years)	≥ 70.0	0.74 (0.67-0.82)	< 0.001	46.1	0.084	0.741
		< 70.0	0.68 (0.40-1.16)	0.155	63.1	0.044	
	Cancer sites	CRC	0.79 (0.67-0.93)	0.004	67.6	0.009	0.173
		Rectal	0.58 (0.44-0.77)	< 0.001	0.0	0.819	
		Colon	0.74 (0.63-0.88)	0.001	0.0	0.419	
	Adjusted level	High	0.80 (0.70-0.91)	0.001	60.7	0.018	0.046
		Low-moderate	0.63 (0.52-0.76)	< 0.001	0.0	0.633	
	Study quality	High	0.80 (0.70-0.92)	0.001	59.7	0.021	0.030
		Moderate	0.63 (0.52-0.75)	< 0.001	0.0	0.684	

### Cancer-specific mortality

**Figure 3:**
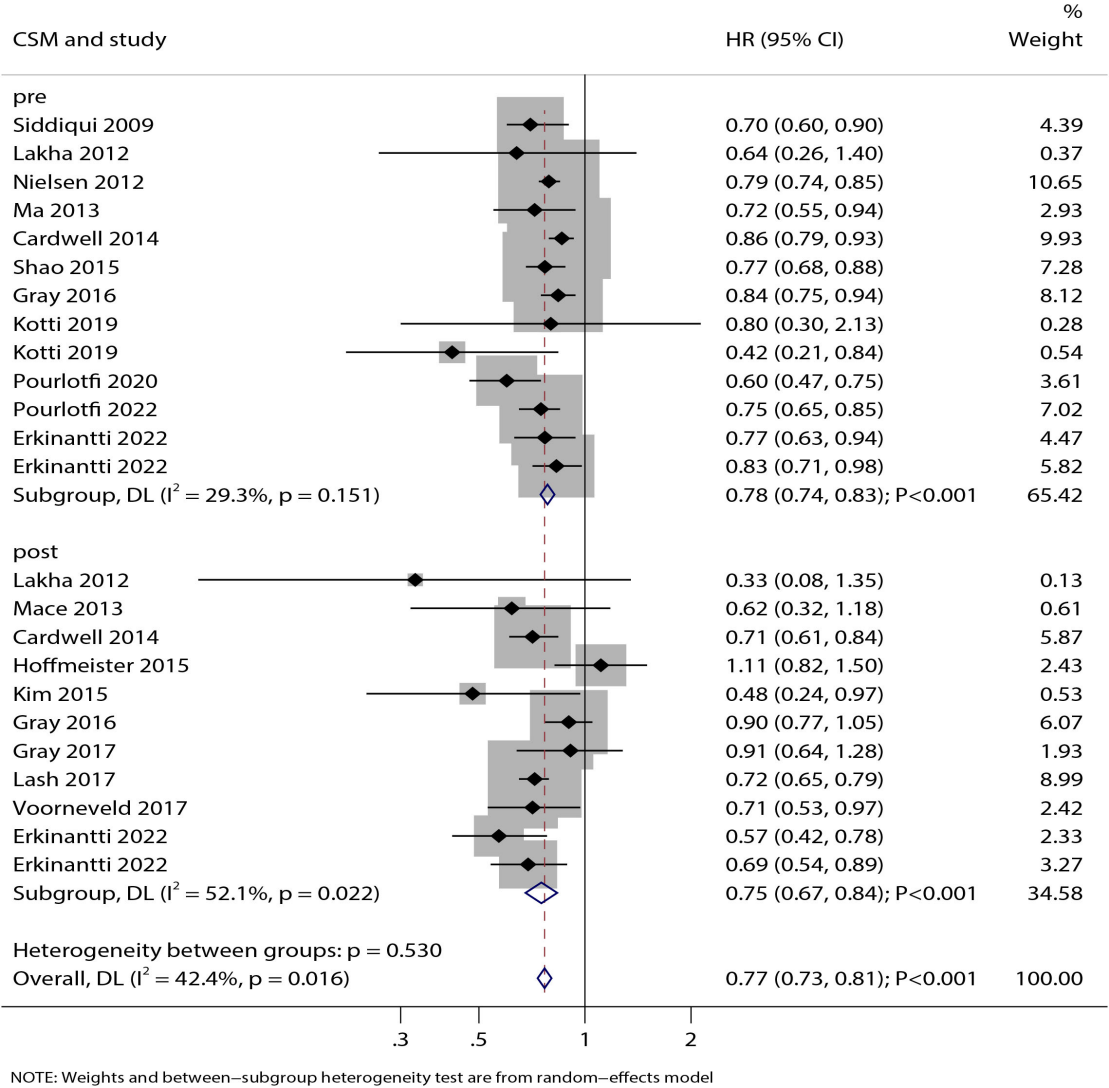
Forest plot of the association between statin use and cancer-specific mortality (CSM) in patients with colorectal cancer. The size of each square represents the study weight, the horizontal line indicates the 95%CI, the vertical line is the null effect line, and the diamond represents the pooled effect size with 95%CI.

The association between statin use and CSM was reported in 12 studies for pre-diagnosis use and 11 studies for post-diagnosis use (**Figure 3**). Statin use was significantly associated with improved CSM (HR: 0.77; 95% CI: 0.73–0.81; P < 0.001). This benefit was observed both for pre-diagnosis statin use (HR: 0.78; 95% CI: 0.74–0.83; P < 0.001) and post-diagnosis statin use (HR: 0.75; 95% CI: 0.67–0.84; P < 0.001). No significant heterogeneity was detected for pre-diagnosis statin use (I² = 29.3%; P = 0.151), whereas significant heterogeneity was observed for post-diagnosis statin use (I² = 52.1%; P = 0.022). Sensitivity analysis indicated that the pooled estimates for the effect of statin use on CSM—both before and after diagnosis—were not driven by any single study (**Supplementary File 2**).

Subgroup analysis showed that pre-diagnosis statin use was consistently associated with improved CSM across all subgroups. In contrast, post-diagnosis statin use showed no significant effect on CSM in the subgroup with mean age <70 years, though significant associations were observed in all other subgroups (**Table 2**). The association between pre-diagnosis statin use and CSM was modified by study design (P = 0.031), while the association for post-diagnosis use was influenced by the extent of covariate adjustment (P = 0.046) and study quality (P = 0.030). Potential publication bias was suggested for pre-diagnosis statin use (Egger’s test P = 0.029; Begg’s test P = 0.100). However, the trim-and-fill adjustment did not alter the significance of this association. For post-diagnosis statin use, no significant publication bias was detected (Egger’s test P = 0.619; Begg’s test P = 0.533) (**Supplementary File 3**).

### Disease-free mortality

**Figure 4:**
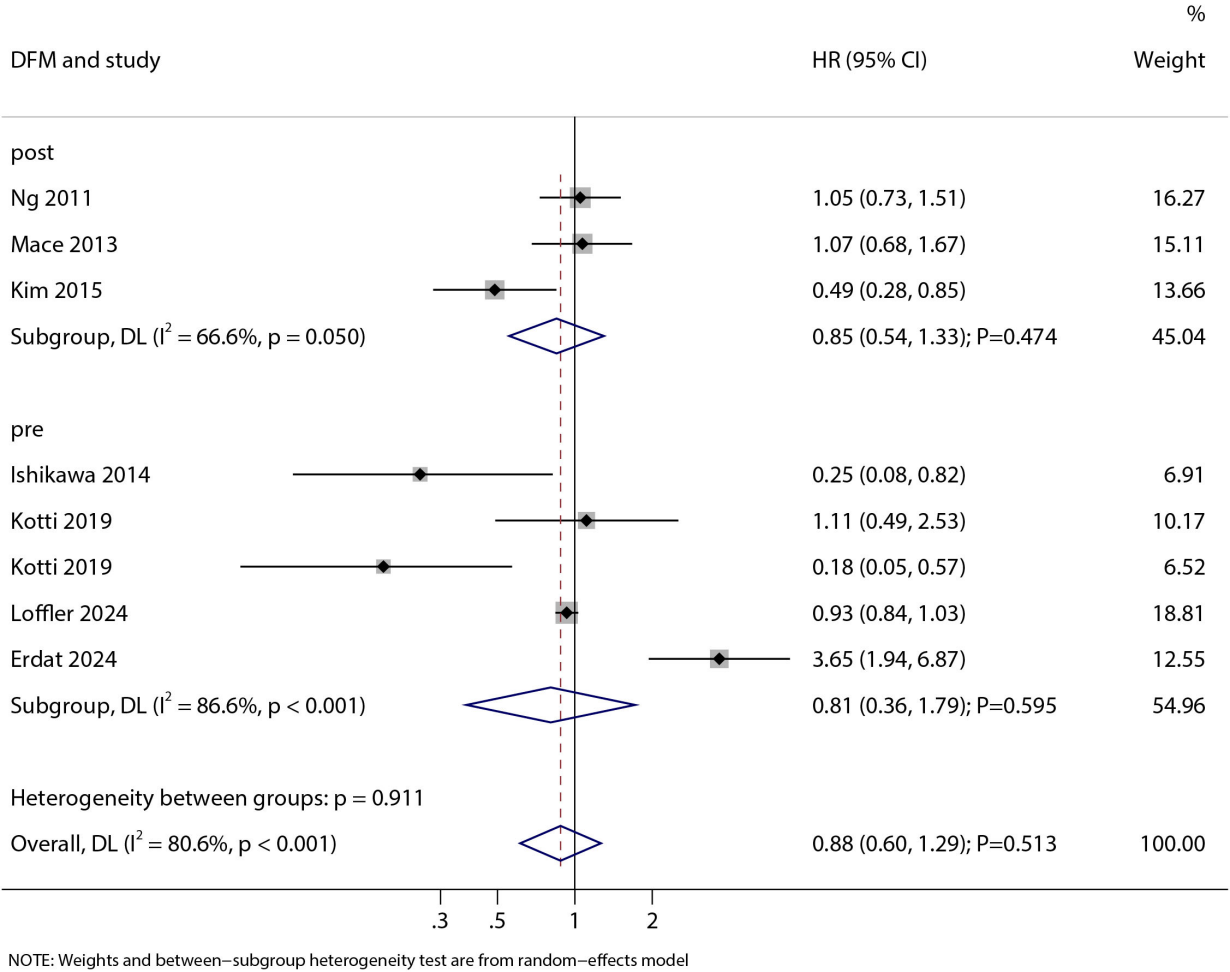
Forest plot of the association between statin use and disease-free mortality (DFM) in patients with colorectal cancer. The size of each square represents the study weight, the horizontal line indicates the 95%CI, the vertical line is the null effect line, and the diamond represents the pooled effect size with 95%CI.

The association between statin use and DFM was reported in 4 studies for pre-diagnosis use and 3 studies for post-diagnosis use (**Figure 4**). Our analysis showed that statin use was not significantly associated with DFM (HR: 0.88; 95% CI: 0.60–1.29; P = 0.513). This lack of significant association was consistent for both pre-diagnosis statin use (HR: 0.81; 95% CI: 0.36–1.79; P = 0.595) and post-diagnosis statin use (HR: 0.85; 95% CI: 0.54–1.33; P = 0.474). Significant heterogeneity was observed for both pre-diagnosis statin use (I² = 86.6%; P < 0.001) and post-diagnosis statin use (I² = 66.6%; P = 0.050).

### Recurrence-free mortality

**Figure 5:**
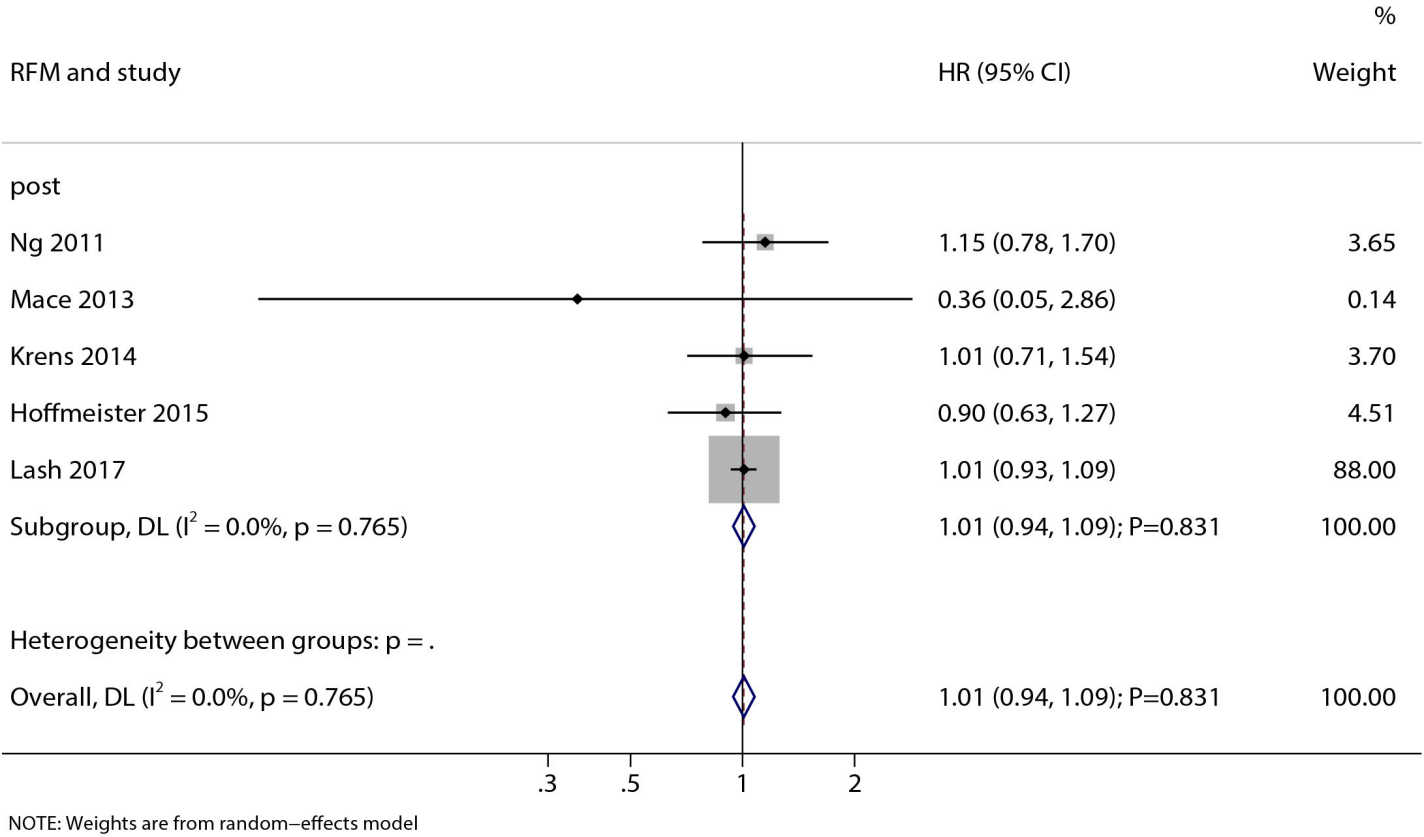
Forest plot of the association between post-diagnostic statin use and recurrence-free mortality (RFM) in patients with colorectal cancer. The size of each square represents the study weight, the horizontal line indicates the 95%CI, the vertical line is the null effect line, and the diamond represents the pooled effect size with 95%CI.

Five studies reported on the association between post-diagnosis statin use and RFM (**Figure 5**). Our analysis indicated that post-diagnosis statin use was not significantly associated with RFM (HR: 1.01; 95% CI: 0.94–1.09; P = 0.831). Furthermore, no significant heterogeneity was observed among the studies (I² = 0.0%; P = 0.765).

## Discussion

Based on a meta-analysis of 25 studies involving 179, 979 patients, this study clarifies the association between statin use and prognosis in CRC patients. Our findings indicate that statin use is significantly associated with reduced ACM and CSM in CRC patients. This benefit was consistently observed with both pre-diagnosis and post-diagnosis statin use, suggesting a potential prognostic value of statins throughout the course of CRC. These results are consistent with previous meta-analyses and further support the “anti-tumor prognostic effect” of statins (12–14). In contrast, no significant association was found between statin use and DFM or RFM.

From a mechanistic perspective, the prognostic benefits of statins may be attributed to multiple factors. First, statins inhibit the mevalonate pathway, thereby reducing cholesterol synthesis required for tumor cell proliferation while downregulating oncogenic signaling pathways such as Ras and Rho, ultimately inducing tumor cell apoptosis (11). Second, the anti-inflammatory properties of statins can suppress inflammatory responses in the tumor microenvironment, reducing angiogenesis and immune suppression, which may delay tumor progression (51, 52). It is worth noting that pre-diagnosis statin use may exert a “chemopreventive” effect by inhibiting malignant transformation in early-stage tumors, whereas post-diagnosis use may directly target residual tumor cells, thereby reducing the risk of recurrence and mortality (53). This could explain the survival benefits observed in both usage scenarios. While our meta-analysis demonstrates a significant association between statin use and improved survival in CRC patients, the underlying mechanisms warrant careful interpretation. The observed reduction in ACM could be partially mediated by the well-established cardiovascular benefits of statins, given that cardiovascular disease is a leading cause of non-cancer mortality in this population. However, the significant and consistent reduction in CSM suggests a potential effect specific to cancer outcomes. This anticancer effect is hypothesized to stem from the mechanisms described above, primarily via mevalonate pathway inhibition. Nonetheless, it is important to acknowledge that the evidence for a direct antitumor effect of statins in human cancers remains associative and is debated in the literature. The survival benefit observed in our analysis may therefore represent a combination of direct antitumor activity, improved cardiovascular health leading to better tolerance of cancer therapies, and the mitigation of cancer-promoting inflammation. Disentangling these contributions in observational data is challenging. In addition, our study did not find a significant association between statin use and DFM or RFM. Possible reasons for this include: (1) the limited number of studies included in the DFM/RFM analysis, along with small sample sizes and high heterogeneity, which may have reduced statistical power; (2) variations in the definitions and evaluation criteria for DFM/RFM across studies, complicating the pooling of results; and (3) statins may primarily reduce cancer-related mortality rather than directly inhibiting tumor recurrence or progression, or a longer follow-up period may be required to observe significant improvements in DFM/RFM.

The heterogeneity analysis in this study revealed population-specific characteristics in the prognostic effects of statins. For ACM, high heterogeneity was observed in both pre-diagnosis and post-diagnosis statin use. In contrast, for CSM, significant heterogeneity was only detected in post-diagnosis use, while pre-diagnosis use showed low heterogeneity. This suggests that the prognostic effect of pre-diagnosis statin use is more stable, potentially due to more consistent baseline characteristics among users. Subgroup analysis further identified key modifiers influencing the prognostic effects of statins: (1) Tumor site significantly modified the ACM benefit of pre-diagnosis use, which may be related to biological differences between colon and rectal tumors (54); (2) Geographic region and patient age influenced the ACM effect of post-diagnosis use, with more pronounced benefits observed in Asian populations and patients aged ≥70 years. This may be attributed to regional treatment patterns and a higher prevalence of cardiovascular comorbidities in older patients, leading to more frequent and prolonged statin use; and (3) The CSM benefit of post-diagnosis use was affected by the extent of covariate adjustment and study quality, indicating that studies with more comprehensive confounding control and higher methodological quality were better able to demonstrate the specific effect of statins while minimizing bias. In response to methodological heterogeneity, subgroup analysis by study design showed that the survival benefit associated with statin use was generally consistent across prospective and retrospective cohorts, supporting the robustness of the primary findings despite the observational nature of all included studies.

The main strengths of this study include: (1) a large sample size (nearly 180, 000 patients) covering multiple regions including Asia, Europe, and North America, with overall high-quality included studies, enhancing the generalizability of the findings; (2) stratified analysis by pre- and post-diagnosis statin use and exploration of multiple modifying factors such as tumor site, age, and geographic region, providing more nuanced clinical evidence; and (3) sensitivity analysis confirming the robustness of the results, with a low risk of publication bias. Meanwhile, this study has several limitations: (1) the most significant limitation is the inherent nature of observational data. Despite multivariate adjustments, residual confounding cannot be ruled out. A major concern is ‘healthy user bias, ‘ whereby patients prescribed statins (typically for cardiovascular indications) may have better overall health literacy, adherence to screening, and engagement with healthcare systems compared to non-users. Furthermore, we lacked data on biological response markers or detailed adherence metrics, which prevents an assessment of a dose-response relationship at the biological level and weakens causal inference; (2) the number of studies on DFM and RFM was limited, with high heterogeneity, requiring cautious interpretation of these results; (3) a significant limitation, as noted in similar meta-analyses, is the lack of detailed and standardized pharmacological data in the primary studies. Information on specific statin types (e.g., hydrophilic vs. lipophilic), doses, and durations of use was inconsistently reported or absent in most included studies, preventing meaningful subgroup or dose-response analyses. This limits our ability to determine the optimal statin regimen for potential prognostic benefit in CRC patients; (4) our analysis focused solely on survival outcomes. None of the included studies systematically reported on statin-associated adverse events (e.g., myopathy, new-onset diabetes) or health-related quality of life (QoL) in CRC patients. The absence of toxicity and QoL data represents a major gap for evaluating the net clinical benefit and for making informed recommendations for adjunctive therapy; and (5) as the analysis was based on published data, there remains a possibility of uncontrolled publication bias.

## Conclusion

This study found that statin use was significantly associated with reduced ACM and CSM in CRC patients, with consistent benefits observed in both pre-diagnosis and post-diagnosis settings. However, no significant association was found between statin use and DFM or RFM. Based on these associative findings, statin therapy could be considered for CRC patients in clinical practice, with individualized decision-making that accounts for factors such as patient age, tumor site, and cardiovascular risk profile. However, the current evidence is insufficient to recommend statins solely as an oncologic adjunct due to the lack of data on toxicity, quality of life, and the unresolved potential for confounding. Future large-scale randomized controlled trials are warranted to determine the optimal timing, dosage, and duration of statin use, to assess patient-reported outcomes and safety, and to validate its effect on the prognosis of CRC patients.

## Data Availability

The original contributions presented in the study are included in the article/**Supplementary Material**. Further inquiries can be directed to the corresponding author.
