# Supplementary material for: Prognostic role of statins in colorectal cancer: a systematic review and meta-analysis
Source: Front Oncol. 2026 Mar 18;16:1763323. doi: 10.3389/fonc.2026.1763323 (PMC13038433; doi:10.3389/fonc.2026.1763323)
Supplement: Supplementary file 2 [file DataSheet1.docx]

**PubMed:**

#1 "Hydroxymethylglutaryl-CoA Reductase Inhibitors"[Mesh] OR statin*[tiab]

#2 "Colorectal Neoplasms"[Mesh] OR "colorectal cancer*"[tiab] OR "colon cancer*"[tiab] OR "rectal cancer*"[tiab]

#3 prognosis[tiab] OR prognos*[tiab] OR surviv*[tiab] OR mortality[tiab] OR recurrence[tiab]

#4 #1 AND #2 AND #3

**EmBase:**

#1 'hydroxymethylglutaryl coenzyme a reductase inhibitor'/exp OR statin*:ti,ab

#2 'colorectal cancer'/exp OR 'colorectal cancer*':ti,ab OR 'colon cancer*':ti,ab ...

#3 'prognosis'/exp OR prognos*:ti,ab OR surviv*:ti,ab OR mortality:ti,ab

#4 #1 AND #2 AND #3

**Cochrane library:**

#1 (statin*):ti,ab,kw

#2 ("colorectal cancer*" OR "colon cancer" OR "rectal cancer"):ti,ab,kw

#3 (prognos* OR surviv* OR mortality OR recurrence):ti,ab,kw

#4 #1 AND #2 AND #3

**Web of Science:**

#1 TS=(statin* OR atorvastatin OR simvastatin OR rosuvastatin OR pravastatin OR lovastatin OR fluvastatin)

#2 TS=("colorectal cancer*" OR "colorectal tumour*" OR "colorectal tumor*" OR "colon cancer*" OR "rectal cancer*")

#3 TS=(prognos* OR surviv* OR mortality OR recurrence OR "disease-free survival" OR "overall survival")

#4 #1 AND #2 AND #3
