# Supplementary material for: Prognostic role of statins in colorectal cancer: a systematic review and meta-analysis
Source: Front Oncol. 2026 Mar 18;16:1763323. doi: 10.3389/fonc.2026.1763323 (PMC13038433; doi:10.3389/fonc.2026.1763323)
Supplement: Supplementary file 4 [file DataSheet3.docx]

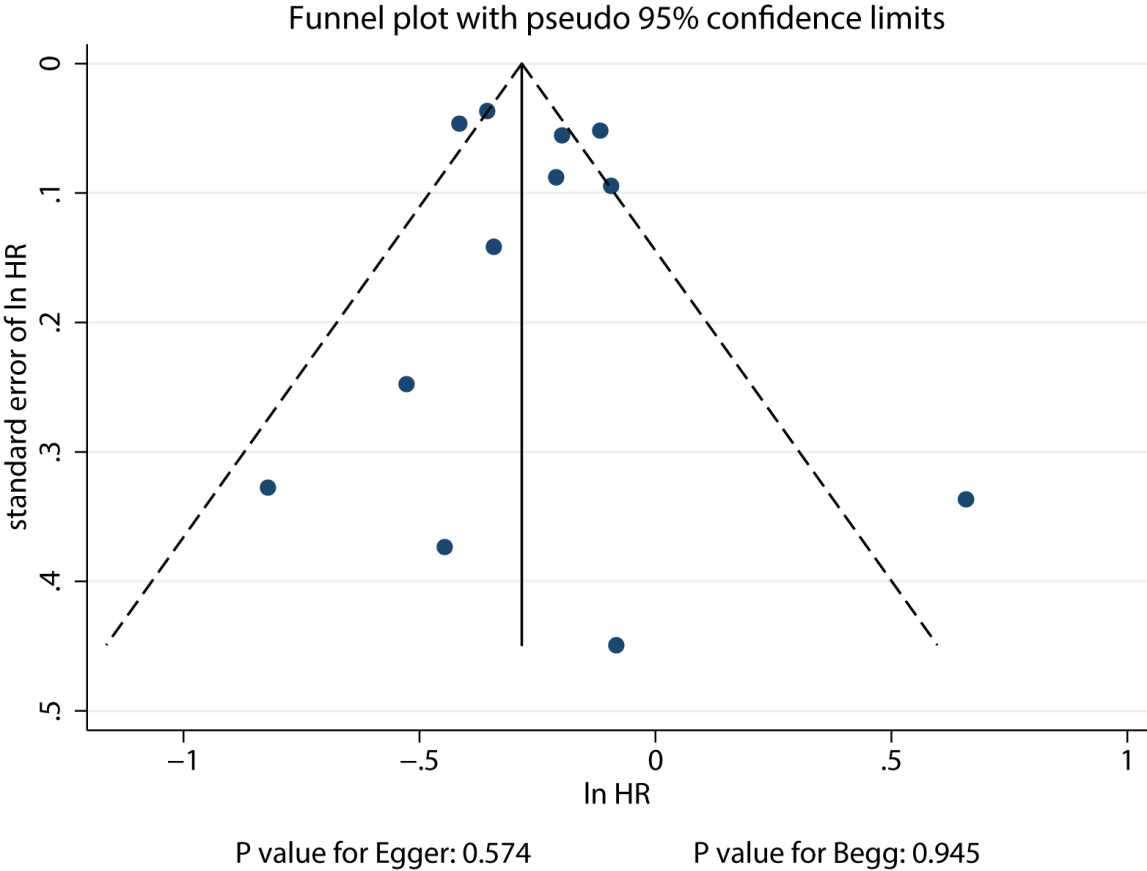


Figure S1. Funnel plot for the association of prediagnosis statin use and ACM


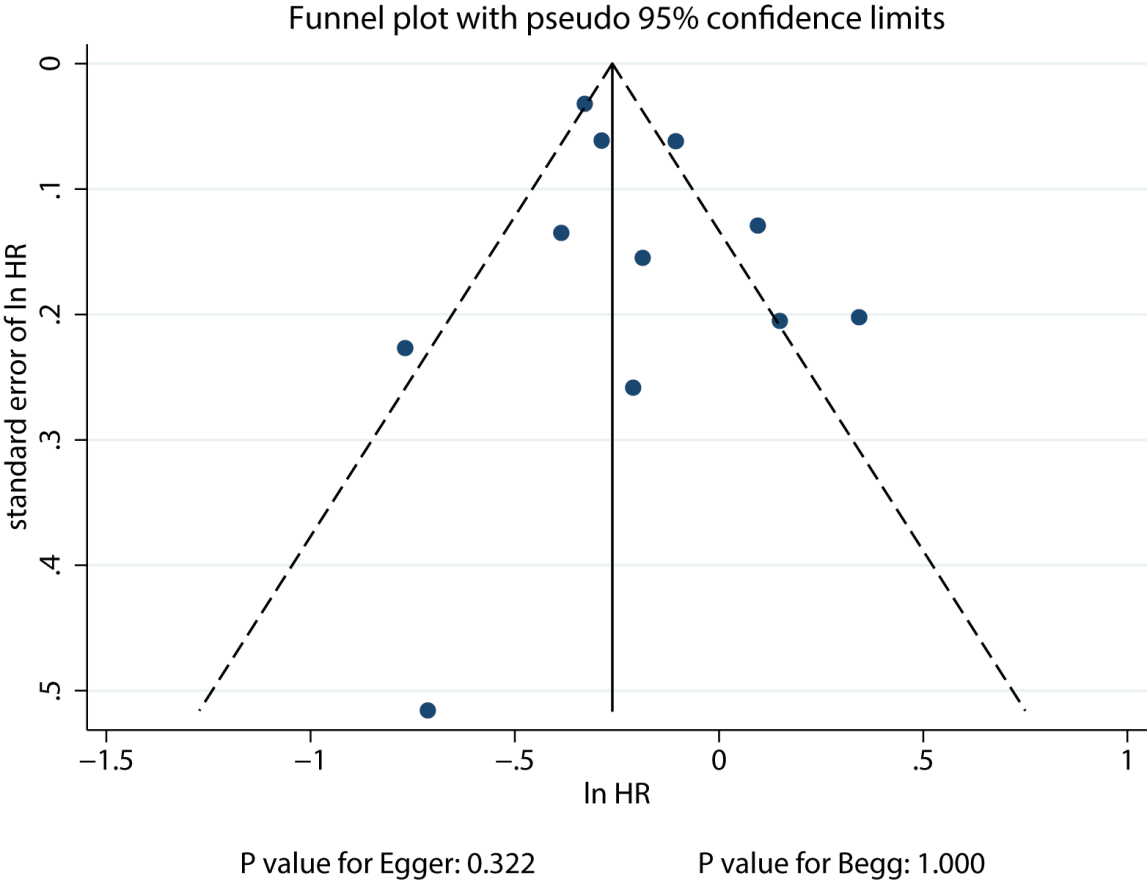


Figure S2. Funnel plot for the association of postdiagnosis statin use and ACM


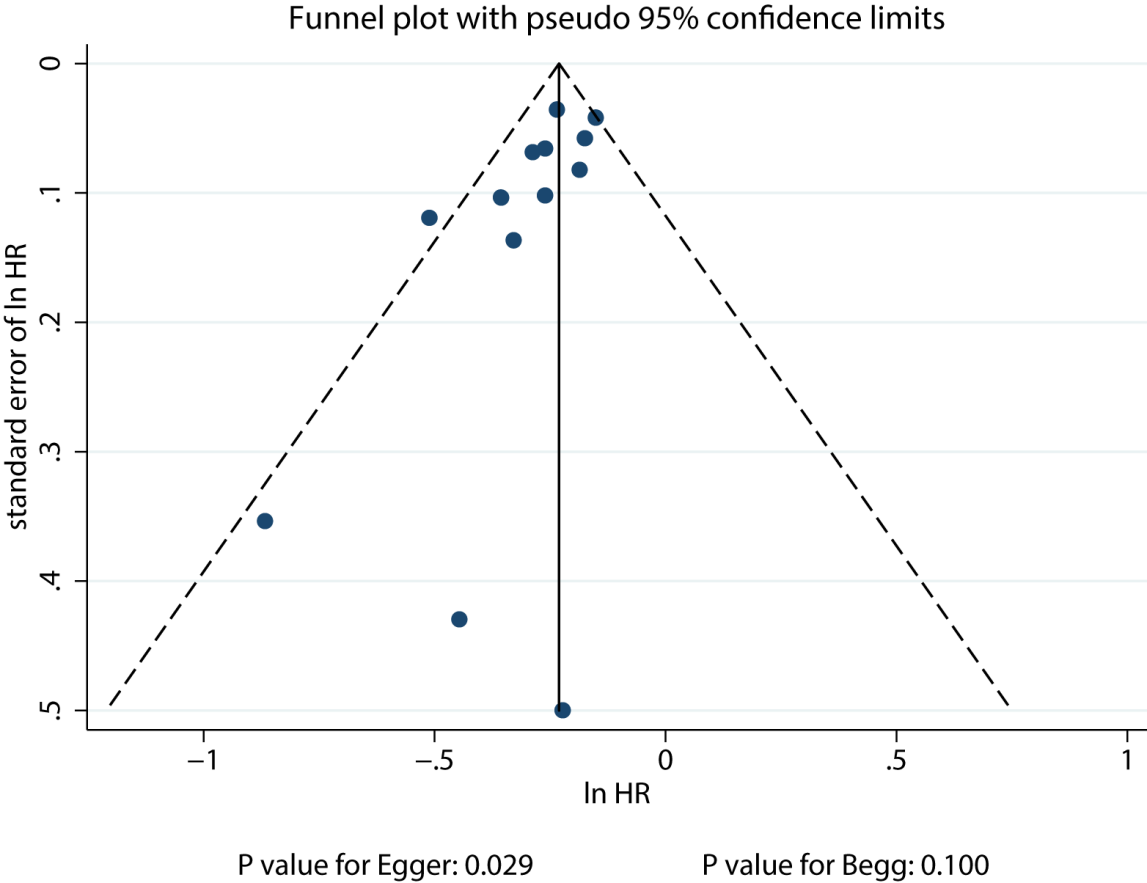


Figure S3. Funnel plot for the association of prediagnosis statin use and CSM


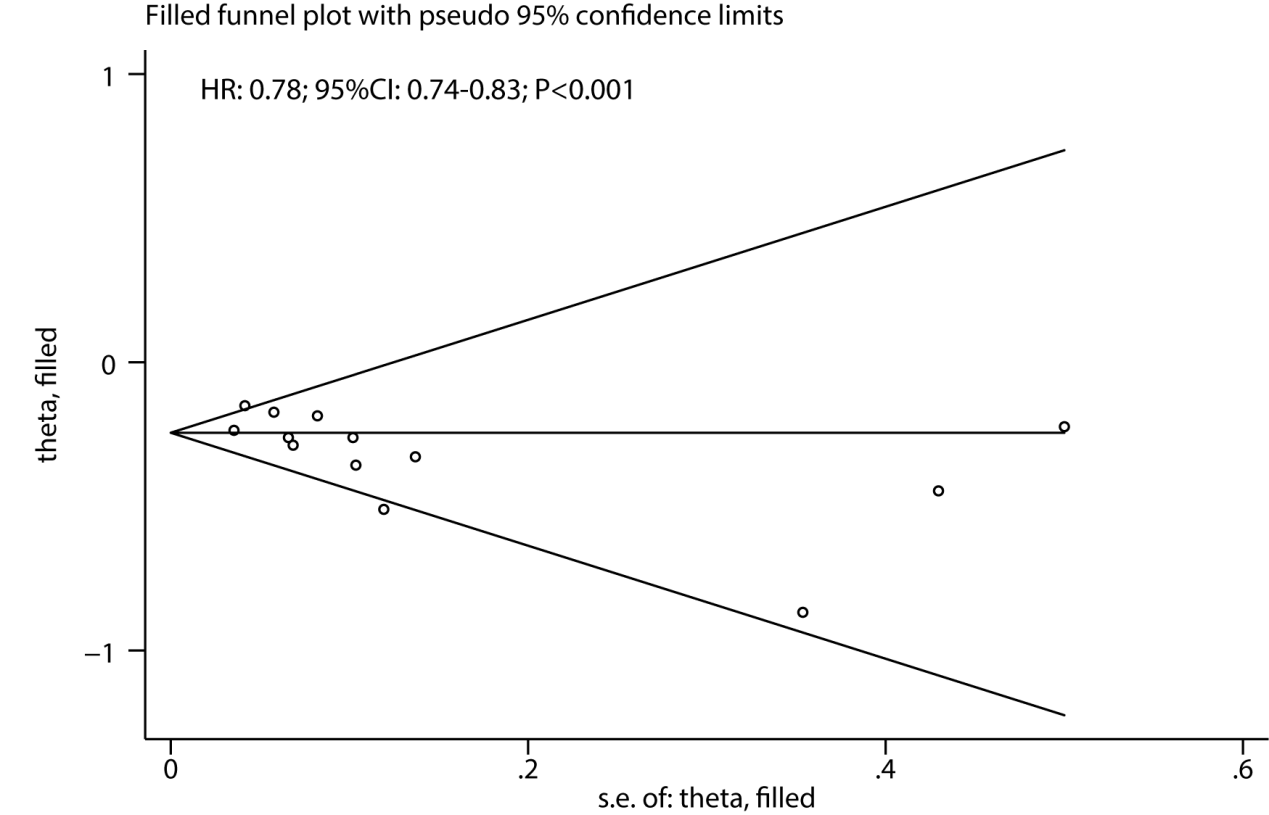


Figure S4. Trim and fill for the association of prediagnosis statin use and CSM


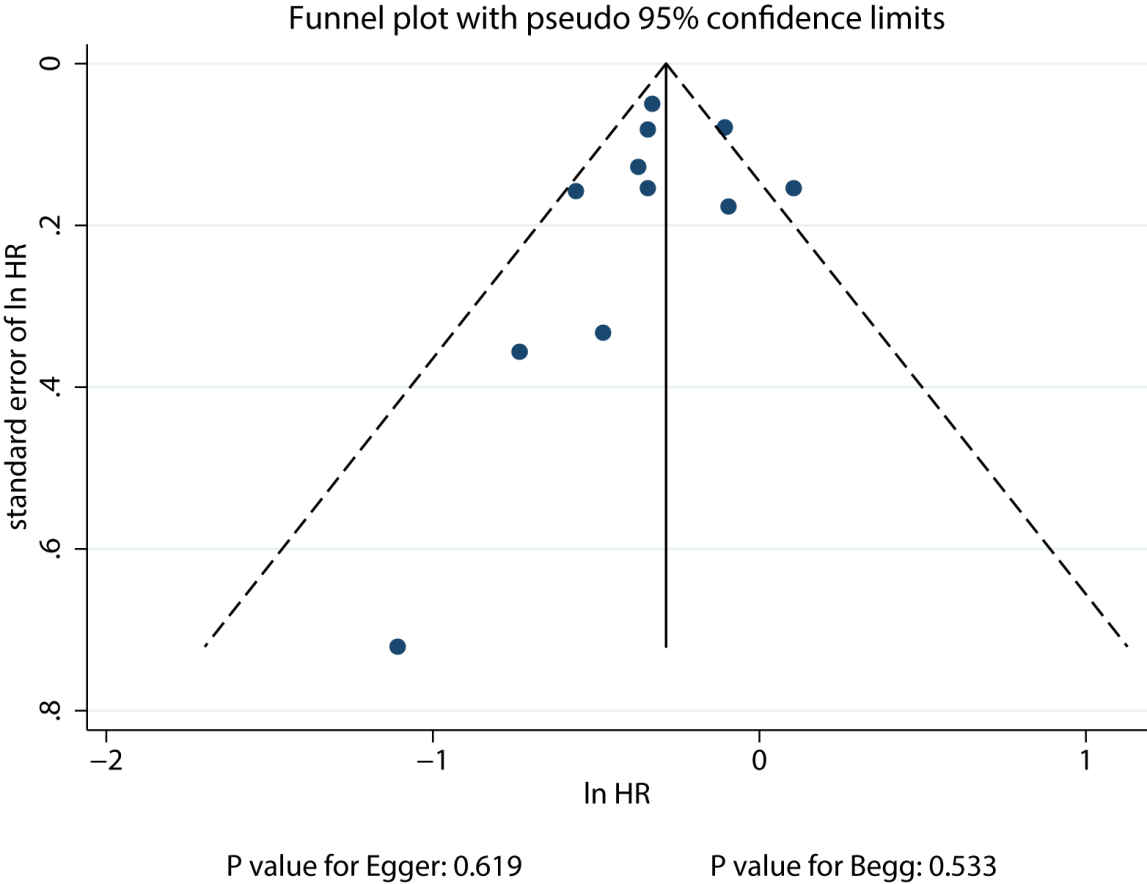


Figure S5. Funnel plot for the association of postdiagnosis statin use and CSM
